# Supplementary material for: Long-term spatial patterns in COVID-19 booster vaccine uptake
Source: Commun Med (Lond). 2025 Jul 1;5:257. doi: 10.1038/s43856-025-00949-w (PMC12215375; doi:10.1038/s43856-025-00949-w)
Supplement: Supplementary file 2 — Supplementary material [file 43856_2025_949_MOESM2_ESM.pdf]

Supplementary Information

Long-term spatial patterns in COVID-19 booster vaccine uptake

Anthony J. Wood, Anne Marie MacKintosh, Martine Stead, Rowland R. Kao

Supplementary Methods 1

The eDRIS data specify for each DZ/age range/sex population group, the total number of individuals to have received exactly one dose, two doses, three doses and four doses. When that is fewer than five individuals, the exact number is not given. To estimate a number of individuals when it is fewer than five, for each population group we fit a two-parameter gamma distribution to the number of individuals to have received exactly n doses, extrapolating to the <5 range. We then estimate the number of individuals for each <5 entry by drawing a value from the distribution.

The random forest model was fit using the *RandomForest* package (version 4.6–14) in *R* (version 4.1.0). Model code is available at `https://git.ecdf.ed.ac.uk/awood310/covid-19-vaccination-analysis-and-p`[illegible]

The ranking of DZs by deprivation are taken from the Scottish Index of Multiple Deprivation, which is publicly available at `https://simd.scot` alongside a detailed methodology of how DZs are ranked. Briefly here, DZs are ranked by deprivation across each of:

- *Access*: an aggregate of measures including access to high-speed broadband, and the average time it takes to travel to public services either by car or public transport;
- *Crime*: the rate of recorded crimes per population;
- *Employment*: relating to the proportion of working adults who are either not working, or in receipt of a type of employment benefit;
- *Housing*: based on the proportion of individuals living in households that are classed as overcrowded, or without central heating;
- *Education*: an aggregate of measures including as the proportion of working age people with no qualifications, and school pupil attendance;
- *Income*: based on the proportion of individuals that are considered *income deprived*, such as being in receipt of Universal Credit;
- *Health*: an aggregate of measures of poor health outcomes including hospital stays due to drug or alcohol misuse, and the number of emergency stays in hospital relative Scotland as a whole.

Supplementary Methods 2

We use uptake in the first booster vaccination rollout, in conjunction with our random forest model informed with SIMD deprivation ranks, to create estimates of longer term distributions, with *lower* nationwide uptake.

We create counterfactual *input* data by making a univariate shift of all deprivation ranks in dataset by some amount Δ. For larger negative values of Δ (thus the ranks being lower, and representative of higher levels of deprivation than in reality), projected uptake is anticipated to fall. A limitation to this is when a counterfactual rank runs outside the range 1–6,976 (e.g., a rank 55 at shift $\Delta = -500$ will be "rank -445"). Random forest models, being a form of stepwise regression, generally perform poorly with data outside the range they are fit against. To address this and project beyond the "floor" rank of each DZ, then, we fit a sigmoid function to each cohort i, for the fit returning uptake U_i as a function of Δ

$$U_i(\Delta) = \frac{1}{1 - \mathrm{e}^{-a_i(\Delta - b_i)}} \, .$$

a_i and b_i are parameters to be fit for each cohort, based on the model fit values for the range of counterfactual Δ values that do not exceed the range 1–6,976. Finally, we shift each fit curve along the y-axis by an amount ϵ_i, such that $U_i(0)$ is the exact observed first booster uptake for cohort i. To avoid scenarios where this shift introduces a modelled uptake greater than 100% or below 0%, we bound $U_i(\Delta) = 0$ if $\left[1 - \mathrm{e}^{-a_i(\Delta - b_i)}\right]^{-1} - \epsilon_i \leq 0$, and 1 if $\left[1 - \mathrm{e}^{-a_i(\Delta - b_i)}\right]^{-1} - \epsilon_i \geq 1$. The $\Delta = 0$ distribution then exactly reproduces that observed for first boosters. We then reduce Δ to generate predicted, lower-uptake distributions.

For about 13% of cohorts the range of valid values for Δ is too narrow to reasonably fit a curve (i.e., instances where a DZ has one very high deprivation rank, and another that is very low). For cohorts where the range is lower than 500 ranks, then, we instead use the average value of parameters a_i and b_i from similar cohorts; having the same age range, sex, DZ deprivation *decile*, and similar first booster uptake.

The underlying random forest model is fit to returning uptake rather than overall uptake. We then calculate the corresponding overall uptake by multiplying relative uptake by overall first dose uptake. By making lower-uptake distributions by decreasing returning uptake, then, we make an implicit assumption that all future doses will be given to individuals that have received at least one vaccination before.

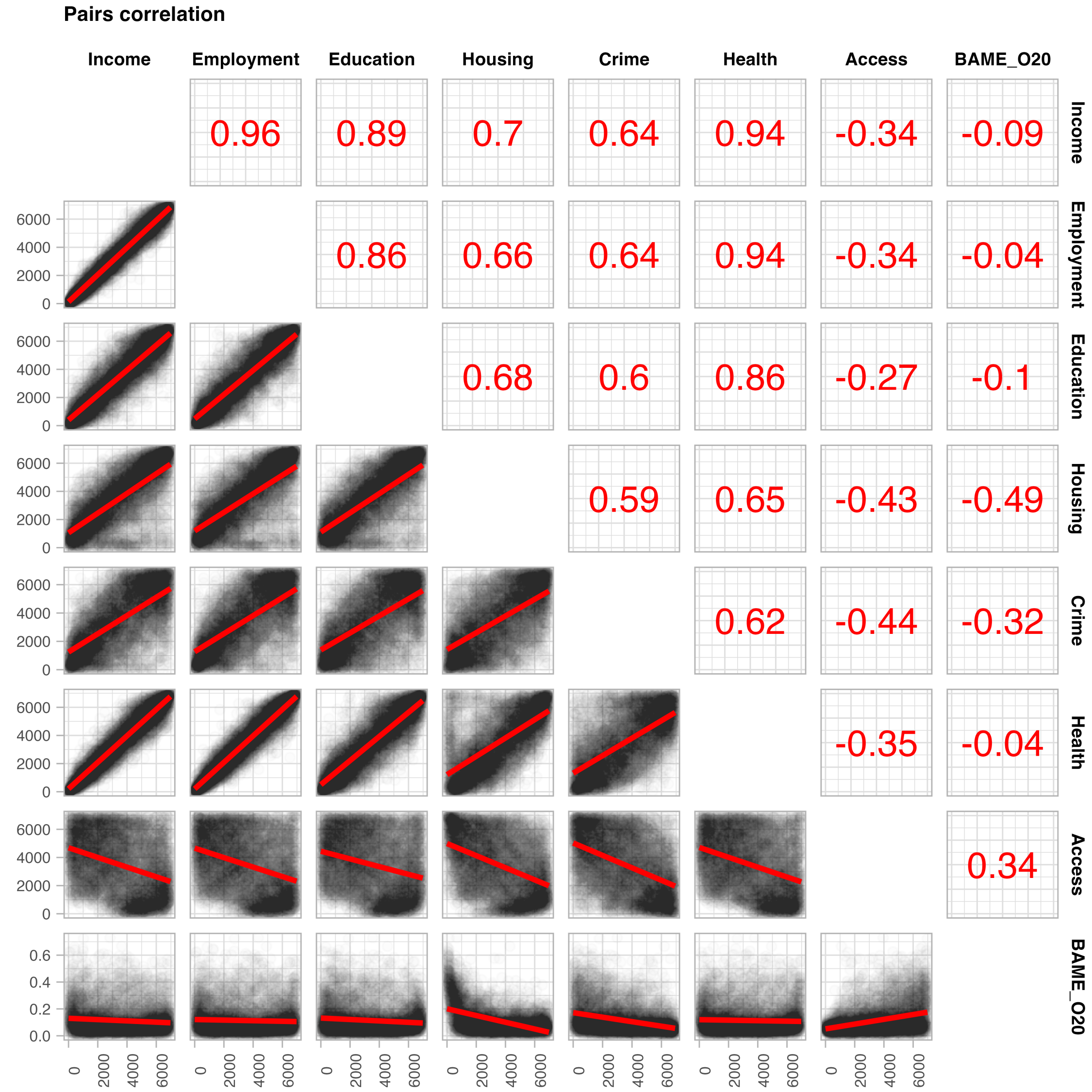

Supplementary Figure 1: Correlation plot (across the 6,976 DZs) between the different deprivation ranks, as well as the proportion of residents aged over 20 belonging to a black or minority ethnicity.

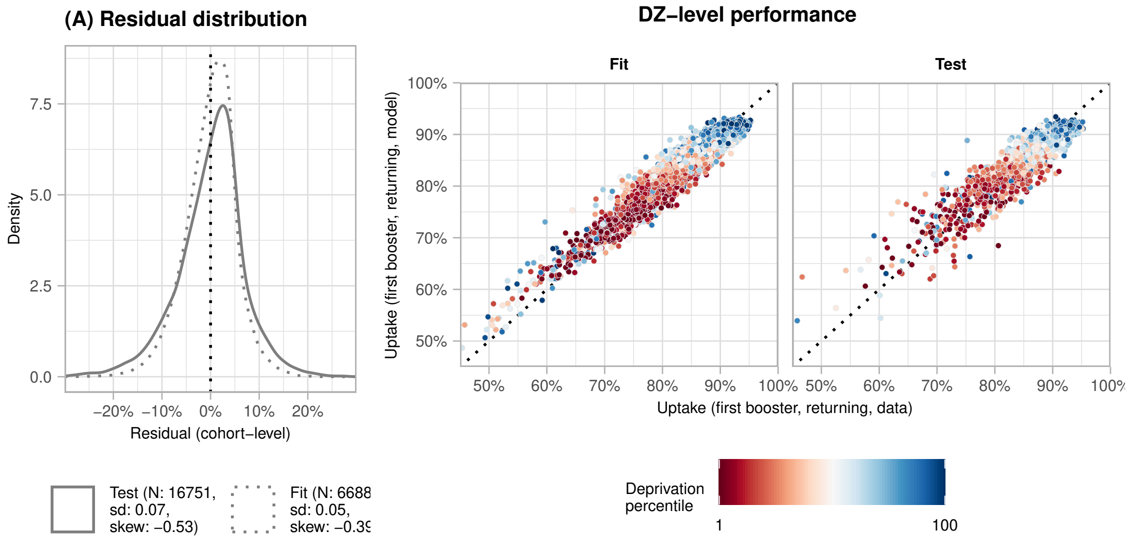

Supplementary Figure 2: Model performance. (**A**) Residual distributions of the fit and test data sets. (**B**) Performance comparing data and fit values for returning uptake at the DZ level, over individual DZs, with deprivation indicated.

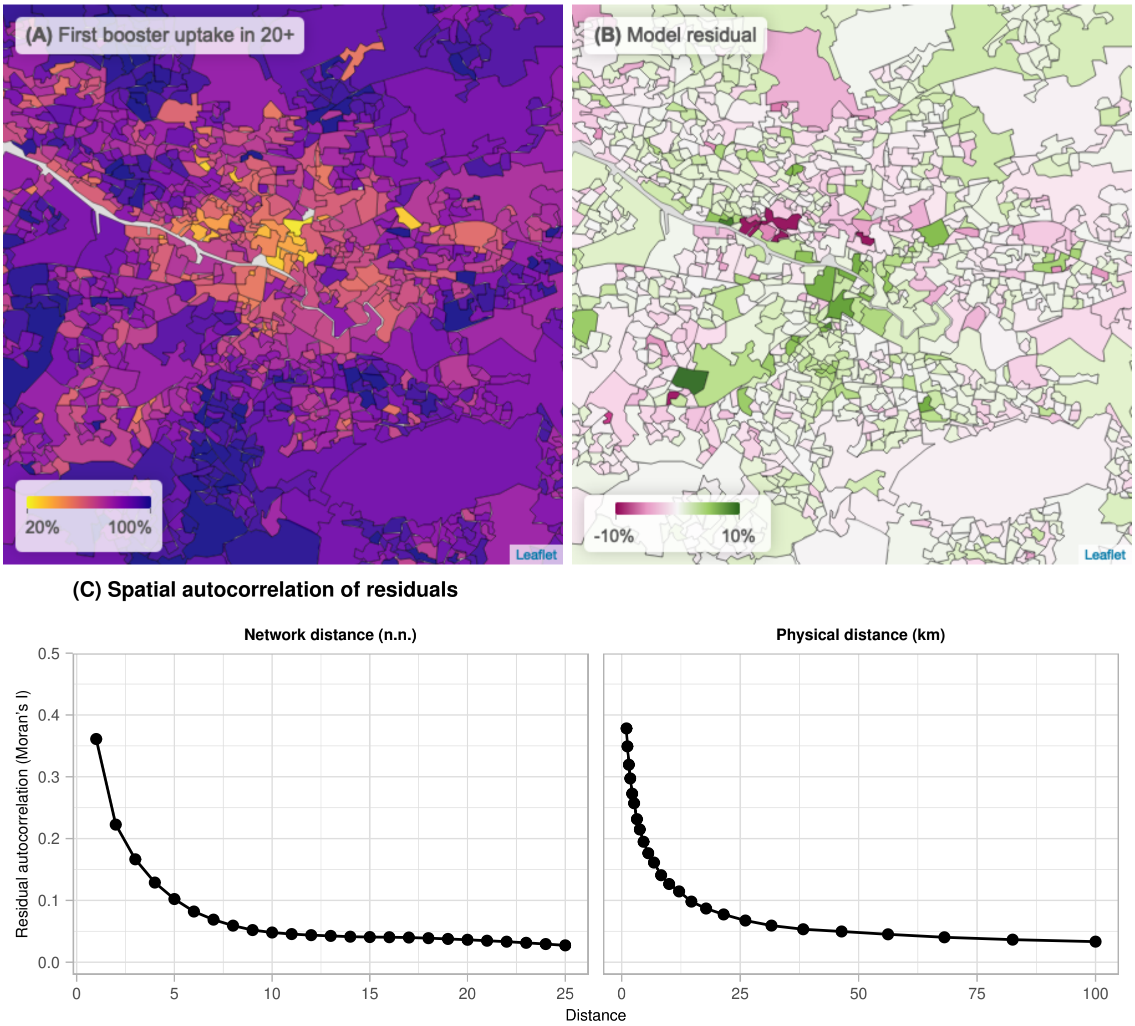

Supplementary Figure 3: (**A**) overall booster uptake across an 18km × 18km area of central Glasgow, and (**B**) corresponding residuals (the difference between actual uptake, and the fit value). Green DZs are those where observed uptake is higher than the fit value, and pink DZs indicate where observed uptake was lower. (**C**) Residual autocorrelation as measured by the Moran's I statistic, comparing autocorrelation between residuals (y-axis) within a certain locus (x-axis). Autocorrelations fall substantially past distances of 5–10km, or within 1–5 nearest neighbours (with nearest neighbours being DZs that share a border).

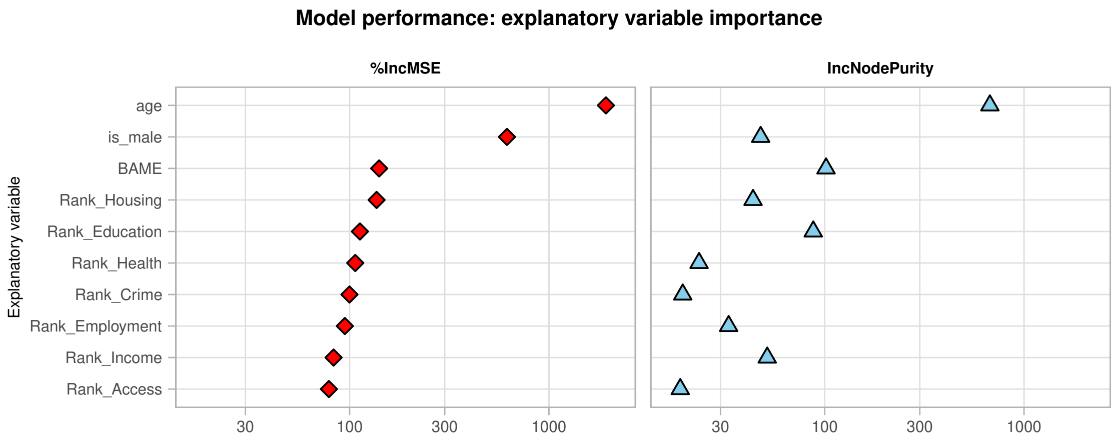

Supplementary Figure 4: Explanatory variable importance output from RF model (MSE loss and node purity).

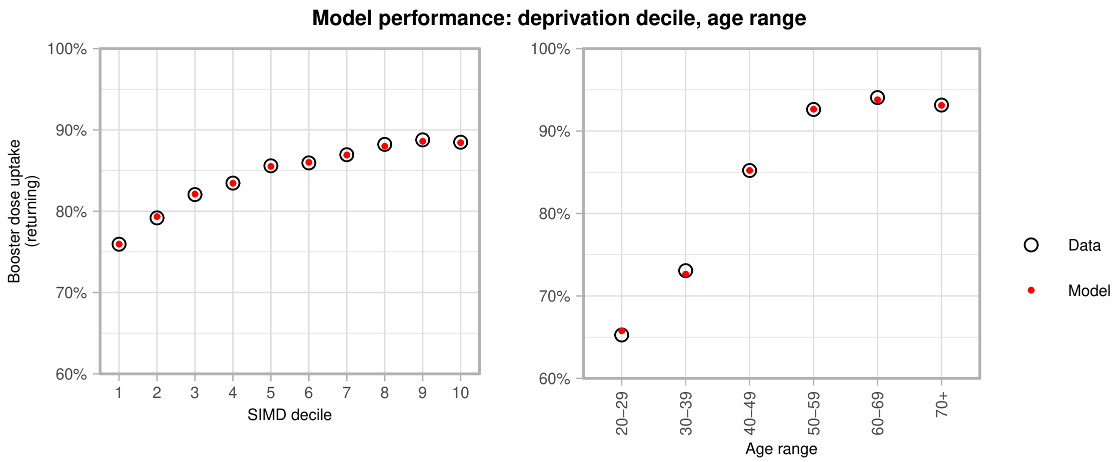

Supplementary Figure 5: Model performance aggregating cohorts over deprivation decile, and age range.

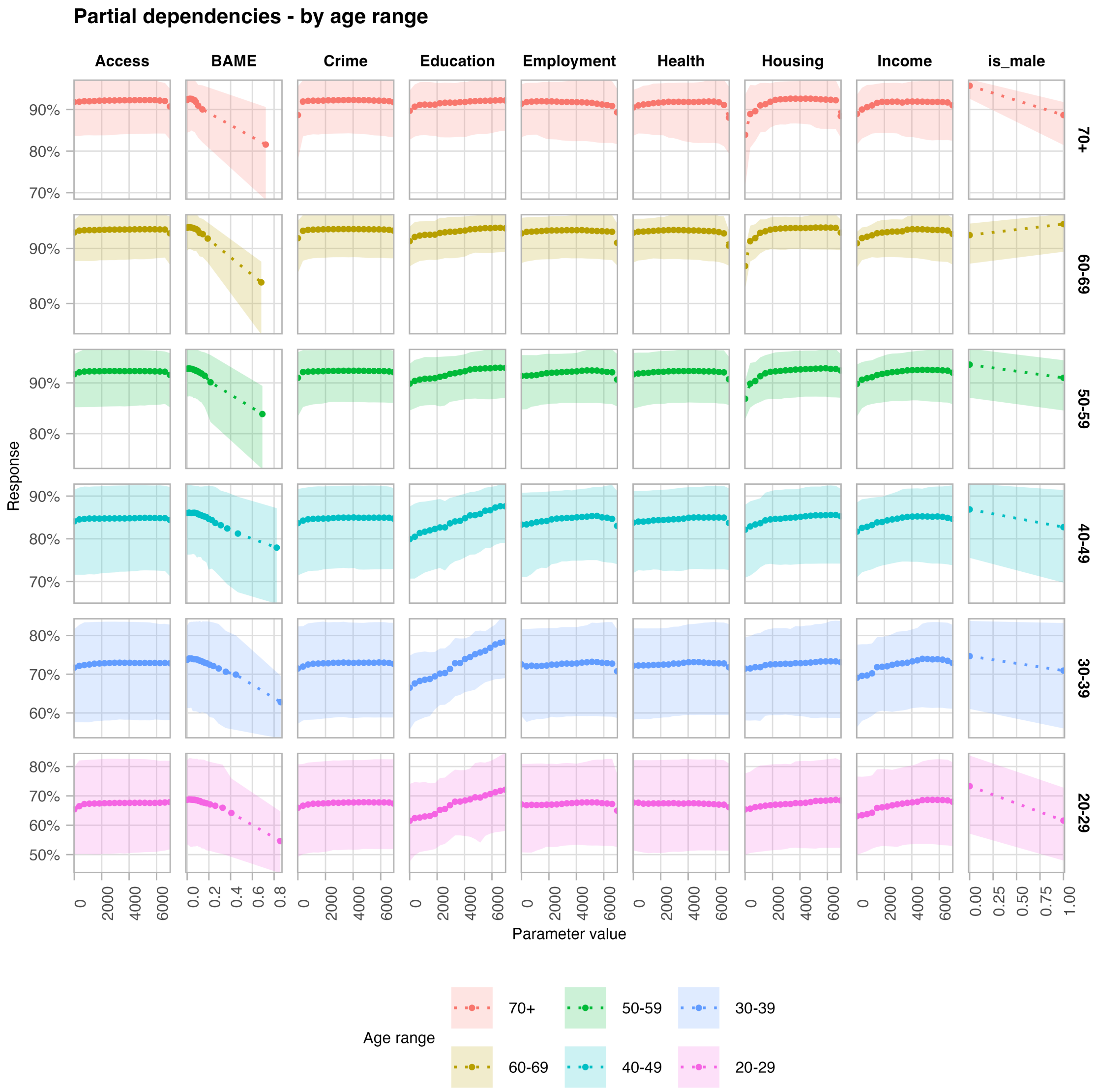

Supplementary Figure 6: Partial dependence plots from the random forest regression divided by age range. This indicates the mean "response" (the model prediction) to each of the particular parameter values, averaged over many different cohorts. The filled region indicates the central 90% of responses.
